# Five-year comparative study of thin-strut rapamycin-eluting bioabsorbable scaffold with metallic drug-eluting stent in porcine coronary artery

**DOI:** 10.3389/fcvm.2022.938519

**Published:** 2022-07-22

**Authors:** Yaokun Liu, Bo Zheng, Bin Zhang, Robert Ndondo-lay, Fangfang Nie, Naijie Tang, Yongsheng Miao, Jianping Li, Yong Huo

**Affiliations:** ^1^Department of Cardiology, Peking University First Hospital, Beijing, China; ^2^Institute of Cardiovascular Disease, Peking University First Hospital, Beijing, China; ^3^Shanghai Biomagic Medical Devices Company Limited, Shanghai, China

**Keywords:** coronary disease, optical coherence tomography, histopathology, bioabsorbable scaffold, swine model

## Abstract

**Objectives:**

Using quantitative coronary angiography (QCA), optical coherence tomography (OCT), histomorphometry, and pharmacokinetics, this study tried to evaluate the safety and efficacy of Biomagic rapamycin-eluting bioabsorbable scaffold (BVS) in non-atherosclerotic porcine coronary arteries.

**Background:**

Biomagic BVS is a new generation of thin-strut bioabsorbable scaffold. We conducted comparative study detailing pathological response, safety and efficacy of Biomagic BVS and the Firebird2 rapamycin-eluting cobalt-based alloy stent (DES) in a porcine coronary artery model. The animals were followed up at 14 days, 1, 3, 6, 12, 18, 24, 30, 36, 42, 48, 54, and 60 months after stent implantation.

**Methods:**

A total of 143 devices (95 Biomagic and 48 Firebird2) were implanted in 2 or 3 main coronary arteries of 76 nonatherosclerotic swine and examined by QCA, OCT, light microscopy, and pharmacokinetics analyses at various time points.

**Results:**

Vascular responses to Biomagic and Firebird2 were largely comparable at all time points, with struts being sequestered within the neointima. The degree of inflammation of both devices was mild to moderate, although the Biomagic score was higher at 14 days to 24 months. However, there was no statistical difference between the two groups except 14 days. At each follow-up time point, the percentage of area stenosis in the Biomagic group was greater than that in the Firebird 2 group, but there was no statistical difference between the two groups at 3 and 12 months. The extent of fibrin deposition was similar between Biomagic and Firebird2, which peaked at 1 month and decreased rapidly thereafter. Pharmacokinetic study showed that coronary tissue sirolimus concentration remained above 2 ng/mg of tissue at 28 day. Histomorphometry showed expansile remodeling of Biomagic-implanted arteries starting after 12 months, and lumen area was significantly greater in Biomagic than Firebird2 at 36 and 42 months. These changes correlated with dismantling of Biomagic seen after 12 months. OCT images confirmed that degradation of Biomagic was complete by 36 months.

**Conclusions:**

Biomagic demonstrates comparable long-term safety to Firebird2 in porcine coronary arteries with mild to moderate inflammation. Although Biomagic was associated with greater percent stenosis relative to Firebird2 within 36 months, expansile remodeling was observed after 12 months in Biomagic with significantly greater lumen area at ≥36 months. Scaffold resorption is considered complete at 36 months.

## Introduction

The bioabsorbable vascular scaffold (BVS) was developed to eliminate some potential risk factors that contributed to long term problems of metallic stents “caging” the coronary artery, such as inflammation (1), endothelium dysfunction (2), in-stent restenosis. Unfortunately, the initial high enthusiasm for Absorb BVS was diminished by its higher incidence of scaffold thrombosis (ST) than that for DES accumulated in 3 years after BVS implantation (3).

From analysis of clinical data, it is learned that most of the ST happened within 6 months after BVS implantation and many ST would have not occurred if small vessels were excluded (3, 4), pretreatment, sizing, post-dilation (PSP) techniques were applied (5), and longer dual-antiplatelet therapy (DAPT) regimen were followed (6, 7). These learnings were confirmed by the Absorb BVS 5-year follow-up studies, which showed that starting from the third year, when the BVS has diminished, target lesion failure (TLF) and ST of BVS have lowered down to the levels of that of DES (8, 9). Additionally, the optimization of scaffold structure may attenuate the potential risk of adverse thrombotic event and lead to favorable long-term outcomes.

We have developed a poly-L-lactide (PLLA)—based BVS (Biomagic) with improved mechanical properties and thinner strut (130–140 μm), anticipating to further improve efficacy and safety (10). The purpose of the present study was to compare the efficacy and safety performance of Biomagic BVS vs. DES in porcine coronary artery model, using quantitative coronary analysis (QCA), optical coherence tomography (OCT), and pathologic analysis, in order to translate the fundamental insight into clinical application in the future.

## Materials and methods

### Study devices

The study device is balloon-expandable Biomagic Rapamycin-Eluting Bioabsorbable Coronary Scaffold System (Biomagic BVS) developed by Shanghai Biomagic Medical Devices Co., Ltd. The scaffold is made of poly (L-lactide) backbone coated with a thin layer of poly (D, L-lactide) mixed with sirolimus in 1:1 ratio. The thickness of scaffold is 130–140 μm. The specification of Biomagic BVS in the study are 3.0 × 8 and 3.0 × 18 mm, with coated drug of 10 μg/mm.

The control device is Firebird2 stent, a balloon-expandable Rapamycin-Eluting Coronary CoCr Stent System made by Shanghai MicroPort Medical (Group) Co., Ltd. The Firebird2 used in the study were 3.0 × 13 mm and 3.0 × 18 mm, coated with sirolimus at 9 μg/mm.

### Animals

This study received protocol approval from the Institutional Animal Care and Use Committee and was conducted in accordance with the regulations and GLP protocols of China National Medical Products Administration (NMPA), with reference to the GLP standards of US FDA.

Seventy-six healthy farm swine ranged 3–4 months of age, weighing 29–43 kg were enrolled for the studies. Animals received standard care and 3 days antiplatelet pretreatment prior the procedure (clopidogrel 75 mg and aspirin 81 mg daily). In each swine, two of the three coronary vessels—left anterior descending (LAD), left circumflex (LCX) and right coronary artery (RCA)—were randomly assigned to implant either Biomagic BVS or Firebird2 DES. The guiding catheter (6F, Cordis) was used as a reference to achieve a 1.1–1.2:1 balloon to artery ratio in order to create a restenosis model. The stent to artery ratio was calculated as: the stent inflated diameter/reference vessel diameter. During the procedure, both QCA and OCT images were acquired prior to and post to the device implantation and saved for further analysis.

After device implantation, the animals were sent to care facilities and normal diet was maintained as well as the daily dual antiplatelet therapy, consisting clopidogrel 75 mg and aspirin 100 mg continued until termination.

At termination, animals were studies with final OCT and/or QCA analysis and euthanized. Vital organs (the heart, liver, lung, spleen, and kidney) were taken for drug content analysis at 14 days, 1 and 3 months. The heart was cut out and coronary vessels flushed with saline and 10% neutral buffered and then kept in 10% neutral buffered formalin for further histopathology and drug content analysis.

### Quantitative coronary analysis

The measurement and statistics of data are completed by Gateway Medical Innovation Center. For all the measurements—both baseline and each follow up—the guiding catheter was used as reference, and standard procedure and method was adopted according to previous literature. The measurements were made from the saved images in DICOM format: reference vessel diameter (RVD), balloon inflated diameter, post-stent minimal lumen diameter (MLD), follow up reference vessel diameter, follow up minimal lumen diameter. The percent diameter stenosis (DS) was calculated as:


DS=[1−(MLD/RVD)×100%

### OCT analysis

The measurement and statistics of data are completed by Gateway Medical Innovation Center. OCT studies were performed using the M2 System (LightLab Imaging, Westford, Mass, USA) and a standard procedure. The OCT images were taken by withdrawn the OCT imaging catheter from the distal to the proximal, across the whole length of the implanted device at 1 mm/s. Artery lumen diameters and lumen areas were measured at three locations of the vessel: distal (2 mm from the distal end of the stent), middle (middle of the stent), and proximal (2 mm from the proximal end of the stent). The minimal lumen diameter (MLD) and minimal lumen area (MLA) are the smallest of the above three respective measurements. The average lumen diameter or lumen area are the averages of the above three respective measurements. The percent area restenosis (AR) was calculated as:


$$\begin{array}{l} \text{AR}=\left[1-MLA\;at\;follow\;up/MLA\;post\;procedure\right]\times 100\% \end{array}$$

### Histopathology analysis

Routine paraffin histology techniques were used for preparing the artery tissue for histopathology analysis: the proximal and distal reference sections were trimmed from the formalin-fixed stented arteries, the trimmed stented arteries were plastic embedded, sectioned at 5–6 μm thickness, and artery sections from proximal and distal ends (taken at 25 and 75% of the stent length) were stained with Hematoxylin and Eosin (H&E). For histopathology analysis, the following cross-sectional areas were directly measured from the tissue sections: the external elastic lamina (EEL), media, internal elastic lamina (IEL), and lumen. The following calculations are made from the direct measurements:


$$\begin{array}{l} \text{Media}=\text{EEL}-\text{IEL}\\ \text{Neointimal Area}=\text{IEL}-\text{Lumen Area}\\ \%\text{Area Stenosis}=\left[1-Lumen\;Area/IEL\right]\times 100\% \end{array}$$

Vessel injury score and neointimal inflammation were scored according to the method by Schwartz et al. (11) and Otsuka et al. (12).

### Pharmacokinetic analysis

Pharmacokinetic analysis was performed from the time of implantation (time 0) to 90 days to evaluate sirolimus elution from the device, sirolimus retention in the coronary tissue and the sounding cardiac muscle tissue, sirolimus concentration in the vital organs and the whole blood. The animal heart was taken at distinct time points and arteries of interest dissected out, cleaned with saline and kept on dry ice. The artery is divided into 3 portions: the stented artery, the portion proximal to stent and the portion distal to stent. The respective cardiac muscle tissues sounding the above artery portions were also dissected out for sirolimus content measurement. The sirolimus content in tissue samples were analyzed by high-performance liquid chromatography (HPLC) (model: Shimadzu LC-30AD). For analytical chromatography a Synergi™ column (2.5 μm, 2 × 50 mm) (Phenomenex, Torrance, CA, USA) was used with the mobile phase (A) water with 0.1% formic acid and 10 mM NH_4_Ac and (B) a solution with 10 mM of NH_4_Ac in H_2_O:ACN:MeOH = 1:3:6. The mobile phase flow rate is 0.5 mL/min. Mass data were acquired and analyzed using Analyst version 1.6.1 (Applied Biosystems-SCIEX, Concord, ON, Canada).

### Statistical analysis

Data are presented as mean ± SD unless otherwise noted. All statistical analysis was performed with SPSS software version 13.0(SPSS Inc., Chicago, IL, USA). No adjustment for multiple implants in an individual animal was made. For QCA and OCT measurements, a linear mixed model was used to evaluate the time and implant effects. The mean differences between comparing devices were tested with ANOVA. For all tests, *p* < 0.05 was considered statistically significant.

## Results

All 76 animals were implanted with 95 Biomagic BVS and 48 Firebird2 DES according to study protocol (see Details in the Supplementary Material). Among these, 2 animals died within 24 h, due to ventricular arrhythmia, leaving 74 animals implanted with 93 BVS and 46 DES in the final analysis. No abnormality was found in preoperative, intraoperative, postoperative and gross anatomy in the remaining 74 animals. The baseline quantitative coronary angiography parameters of pre-implantation MLD and post-implantation MLD of the BVS and Firebird2 were comparable and further confirms successful implantation.

### QCA analysis

QCA results from 14 days to 60 months after device implantation were summarized in Table 1. The balloon-to-artery ration between two groups are similar in all but the 1- and 6-month groups, where the balloon-to-artery ratio for the Biomagic BVS group were 1.0 ~ 1.1 vs. 1.1 ~ 1.2 for the Firebird2 groups.

**Table 1:** Quantitative coronary angiography.

	**14 d**	**1 m**	**3 m**	**6 m**	**12 m**	**18 m**	**24 m**	**30 m**	**36 m**	**48 m**	**54 m**	**60 m**
**Balloon-to-artery ratio**												
Biomagic	1.1 ± 0.1	1.0 ± 0.1	1.1 ± 0.1	1.1 ± 0.1	1.1 ± 0.1	1.1 ± 0.1	1.1 ± 0.1	1.1 ± 0.1	1.1 ± 0.1	1.1 ± 0.1	1	1.1
Firebird2	1.1 ± 0.0	1.1 ± 0.1	1.1 ± 0.1	1.2 ± 0.1	1.1 ± 0.0	1.1 ± 0.0	1.1 ± 0.1	1.1 ± 0.0	1.2 ± 0.1	1.3	/	1.3
*P-value*	0.161	0.046	0.214	0.011	0.240	0.327	0.033	0.481	0.077	/	/	/
**Preimplant mean luminal diameter, mm**												
Biomagic	2.7 ± 0.1	2.6 ± 0.1	2.5 ± 0.1	2.5 ± 0.1	2.7 ± 0.2	2.7 ± 0.1	2.3 ± 0.7	2.2 ± 0.8	2.2 ± 0.7	2.5 ± 0.2	1.8 ± 1.0	2.3
Firebird2	2.7 ± 0.1	2.7 ± 0.1	2.6 ± 0.2	2.6 ± 0.2	2.8 ± 0.1	2.7 ± 0.2	2.7 ± 0.2	2.6 ± 0.1	2.5 ± 0.1	2.4	2.4	2.4
*P-value*	0.597	0.483	0.138	0.065	0.471	1.000	0.466	0.854	0.829	0.793	0.667	/
**Postimplant mean luminal diameter, mm**												
Biomagic	2.8 ± 0.1	2.7 ± 0.1	2.7 ± 0.1	2.7 ± 0.1	3.0 ± 0	3.0 ± 0.1	2.5 ± 0.8	2.4 ± 0.8	2.4 ± 0.8	2.6 ± 0.1	2.0 ± 1.1	2.7
Firebird2	3.0 ± 0.1	3.1 ± 0.1	3.0 ± 0.2	3.0 ± 0.2	3.2 ± 0.1	3.0 ± 0.2	3.0 ± 0.2	3.1 ± 0.1	2.9 ± 0.2	2.7	2.7	2.7
*P-value*	0.003	0.000	0.003	0.001	0.022	0.432	0.026	0.927	0.384	0.645	0.667	/
**Follow-up mean luminal diameter, mm**												
Biomagic	2.2 ± 0.2	2.0 ± 0.2	2.0 ± 0.2	2.0 ± 0.3	2.6 ± 0.3	2.7 ± 0.4	2.7 ± 0.9	2.7 ± 1.0	2.8 ± 1.0	3.2 ± 0.3	2.2 ± 1.1	2.8
Firebird2	2.8 ± 0.2	2.4 ± 0.4	2.2 ± 0.4	2.6 ± 0.3	3.0 ± 0	2.6 ± 0.3	2.7 ± 0.3	2.7 ± 0.1	2.5 ± 0.2	2.4	2.9	2.9
*P-value*	0.000	0.032	0.123	0.001	0.123	0.408	0.369	0.386	0.004	0.840	0.333	/
**Late lumen loss, mm**												
Biomagic	0.6 ± 0.2	0.7 ± 0.2	0.7 ± 0.3	0.7 ± 0.3	0.5 ± 0.4	0.1 ± 0.4	−0.2 ± 0.4	−0.2 ± 0.9	−0.3 ± 0.4	−0.6 ± 0.3	−0.2 ± 0.3	/
Firebird2	0.2 ± 0.2	0.7 ± 0.4	0.8 ± 0.4	0.4 ± 0.2	0.2 ± 0.1	0.3 ± 0.1	0.3 ± 0.2	0.3 ± 0.2	0.4 ± 0.3	0.3	−0.2	/
*P-value*	0.001	0.762	0.889	0.032	0.292	0.232	0.042	0.226	0.003	0.404	0.821	/
**Percent diameter stenosis, %**												
Biomagic	20.9 ± 8.0	26.7 ± 7.9	26.8 ± 10.4	24.8 ± 10.9	14.2 ± 11.1	7.6 ± 13.3	−7.6 ± 18.1	−8.5 ± 18.3	−12.5 ± 18.2	−22.6 ± 8.6	−5.4 ± 12.9	−3.7
Firebird2	6.3 ± 5.3	22.2 ± 12.6	25.4 ± 11.5	12.8 ± 6.7	4.7 ± 1.5	14.8 ± 8.5	10.0 ± 6.1	12.8 ± 4.3	11.2 ± 10.1	11.11	−7.41	−7.41
*P-value*	0.000	0.398	0.784	0.013	0.279	0.247	0.053	0.607	0.004	0.149	0.959	/

*Biomagic indicates Biomagic rapamycin-eluting bioresorbable coronary scaffold system. P-value for Biomagic vs. Firebird2 at each time point. d, day; m, month*.

The pre-implant mean lumen diameter (LD) between the two groups are similar in all time points. The post-implant mean LDs were significantly smaller in Biomagic BVS group compared to control group, especially in early time points (14-day to 12-month) and 24-month groups, but not in later (30- to 60-month) groups.

Late lumen loss (LLL) in most time points were similar between two groups, except that (1) in the 14-day and 6-month group, Biomagic BVS had significantly greater LLL than that for Firebird2 DES, with 0.6 ± 0.2 mm vs. 0.2 ± 0.2 mm (*p* = 0.001), and 0.7 ± 0.3 mm vs. 0.4 ± 0.2 mm (*p* = 0.032), respectively; (2) from 18 month on, Biomagic BVS groups showed a tendency of reducing LLL and after 24 month to reach negative LLL (from −0.2 to −0.6 mm on average); (3) significantly smaller LLL in Biomagic BVS groups than those for Firebird2 DES were found in 24- and 36-month groups, with −0.2 ± 0.4 mm vs. 0.3 ± 0.2 mm (*p* = 0.042), and −0.3 ± 0.4 mm vs. 0.4 ± 0.3 mm (*p* = 0.003), respectively.

Percent diameter stenosis (DS) were (1) generally greater for Biomagic BVS than those for Firebird2 DES for all time points up to 18-month, with 14-day and 6-month groups significantly higher for BVS, with 20.9 ± 8.0% vs. 6.3 ± 5.3% (*p* = 0.000) and 24.8 ± 10.9% vs. 12.8 ± 6.7% (*p* = 0.013), respectively; (2) from 18 month on, BVS groups showed lowering tendency of DS and after 24 month to reach negative DS (from −3.7 to −22.6%); (3) significantly smaller DS in BVS groups than those for DES were found in 36-month, with −12.5 ± 18.2% vs. 11.2 ± 10.1% (*p* = 0.004).

### OCT analysis

**Figure 1:**
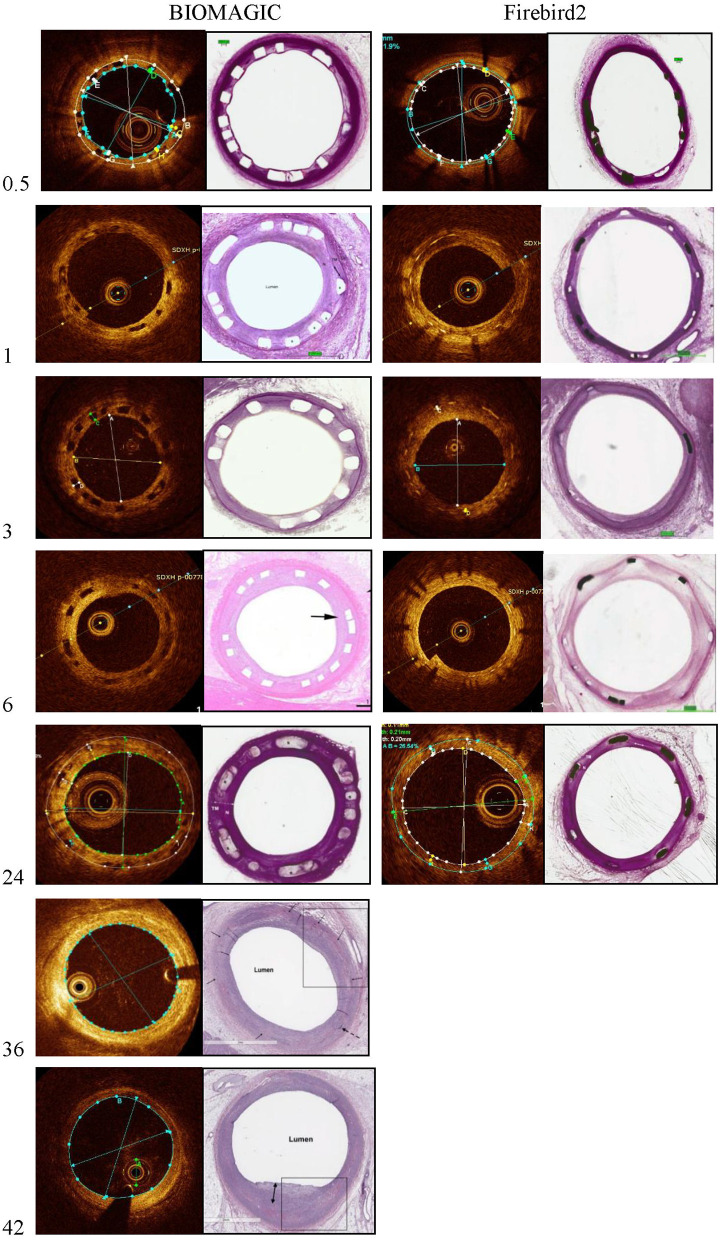
Representative photomicrographs and OCT still frames of Biomagic and Firebird2-implanted porcine coronary arteries evaluated from 1 to 42 months.

Representative OCT images of BVS and DES at 14 days, 1, 3, 6, 12, 24, 36, and 42 months are shown in Figure 1. OCT results are summarized in Table 2. The mean scaffold diameter for Biomagic BVS was smallest in 3-month (2.90 ± 0.20 mm), but increased from 6-month (3.20 ± 0.20 mm) to above 3.9 mm after 24-month. In contrast, the mean DES diameter remained relatively stable from 14-day to 18-month, became slightly smaller from 24-month onward. The divergent changes in diameter for BVS and DES resulted in BVS having significantly smaller diameters in early times (1-, 3-, and 6-month), similar diameter in mid times (12- and 18-month), but significantly larger diameters after 24-month.

**Table 2:** OCT analysis.

	**14 d**	**1 m**	**3 m**	**6 m**	**12 m**	**18 m**	**24 m**	**30 m**	**36 m**
**Follow-up mean scaffold diameter, mm**									
Biomagic	3.12 ± 0.37	3.30 ± 0.10	2.90 ± 0.20	3.20 ± 0.20	3.50 ± 0.30	3.51 ± 0.31	3.99 ± 0.61	3.91 ± 0.09	3.95 ± 1.64
Firebird2	3.30 ± 0.31	3.50 ± 0.30	3.30 ± 0.30	3.50 ± 0.20	3.37 ± 0.08	3.23 ± 0.42	3.13 ± 0.24	2.91 ± 0.24	2.91 ± 0.24
*p*-value	0.287	0.045	0.001	0.035	0.408	0.207	0.000	0.002	0.002
**Follow-up mean luminal diameter, mm**									
Biomagic	2.65 ± 0.35	2.78 ± 0.15	2.20 ± 0.34	2.49 ± 0.25	2.86 ± 0.32	2.69 ± 0.35	3.18 ± 0.22	3.19 ± 0.07	3.26 ± 0.39
Firebird2	3.08 ± 0.30	3.08 ± 0.24	2.71 ± 0.40	3.07 ± 0.27	2.88 ± 0.06	2.83 ± 0.37	2.79 ± 0.15	2.74 ± 0.25	2.70 ± 0.21
*p*-value	0.024	0.012	0.013	0.000	0.937	0.518	0.009	0.018	0.017
**Follow-up mean scaffold area, mm^2^**									
Biomagic	7.63 ± 1.73	6.5 ± 1.1	5.86 ± 1.04	8.25 ± 0.85	7.88 ± 2.15	9.74 ± 1.74	12.06 ± 1.49	12.01 ± 0.64	13.84 ± 2.76
Firebird2	8.36 ± 1.44	6.7 ± 1.4	7.89 ± 1.06	9.17 ± 1.08	6.58 ± 1.43	8.34 ± 2.09	7.73 ± 1.14	7.43 ± 1.24	6.75 ± 1.13
*p*-value	0.379	0.718	0.016	0.104	0.244	0.502	0.000	0.000	0.002
**Follow-up mean luminal area, mm^2^**									
Biomagic	5.81 ± 1.55	8.00 ± 1.98	3.14 ± 1.17	5.01 ± 0.83	8.65 ± 2.48	5.83 ± 1.46	7.77 ± 1.20	7.86 ± 0.29	8.55 ± 2.08
Firebird2	7.03 ± 1.57	9.35 ± 0.98	4.37 ± 1.05	6.95 ± 1.08	7.33 ± 1.01	6.39 ± 1.62	6.11 ± 0.63	6.19 ± 1.05	5.83 ± 0.81
*p*-value	0.070	0.060	0.120	0.000	0.280	0.500	0.030	0.030	0.010
**Follow-up minimal luminal area, mm^2^**									
Biomagic	2.23 ± 1.64	3.39 ± 2.31	2.11 ± 1.17	2.81 ± 0.77	3.7 ± 2.33	3.34 ± 1.43	5.61 ± 1.07	6.52 ± 0.54	5.24 ± 1.92
Firebird2	4.24 ± 1.47	8.31 ± 0.98	2.87 ± 1.05	5.11 ± 1.12	6.15 ± 1.02	4.04 ± 1.37	4.85 ± 0.67	5.04 ± 0.82	4.43 ± 0.73
*p*-value	0.192	0.138	0.117	0.001	0.458	0.311	0.022	0.043	0.008
**Percent area stenosis, %**									
Biomagic	28.50 ± 5.72	36.00 ± 7.50	44.10 ± 9.00	43.20 ± 7.50	38.21 ± 7.79	44.57 ± 5.85	36.03 ± 15.43	36.35 ± 3.11	43.64 ± 18.31
Firebird2	14.63 ± 4.38	27.00 ± 9.40	41.70 ± 11.70	26.20 ± 8.60	28.95 ± 5.73	24.69 ± 7.43	22.41 ± 4.82	18.93 ± 1.86	14.37 ± 2.28
*p*-value	0.000	0.048	0.633	0.000	0.196	0.000	0.005	0.001	0.000

*Biomagic indicates Biomagic rapamycin-eluting bioresorbable coronary scaffold system. Firebird2 indicates Firebird2 rapamycin-eluting cobalt-based alloy stent. P-value for Biomagic vs. Firebird2 at each time point. d, day; m, month*.

The mean lumen diameter for Biomagic BVS was smallest in 3-month (2.20 ± 0.34 mm), but increased from 6-month (2.49 ± 0.25 mm) to above 3.18 ± 0.22 mm after 24-month. The mean lumen diameter for DES remained relatively stable from 14-day to 6-month, with a slight drop in 3-month (2.70 ± 0.40 mm), became gradually smaller from 12-month (2.88 ± 0.06 mm) to 36-month (2.70 ± 0.21 mm). The divergent changes in diameter for BVS and DES resulted in BVS having significantly smaller lumen diameters in 4 early times (14 day, 1-, 3-, and 6-month), similar lumen diameter in mid times (12- and 18-month), but significantly larger diameters after 24-month.

The percent area restenosis (%AS) were significantly higher for the Biomagic BVS group than that for the Firebird2 DES group in most of the time points, except the 3-month and 12-month. However, the lumen diameter and scaffold diameter of the Biomagic became larger with time. Meanwhile, the lumen area enlarged after 18 months and the effective lumen was larger in Biomagic as compared. This trend is similar to previous studies on the percentage of stenosis in the histological research of Absorb and XIENCE V (13). And OCT data showed that most BVS had been completely degraded at 3-year follow-up.

### Histological analysis

**Figure 2:**
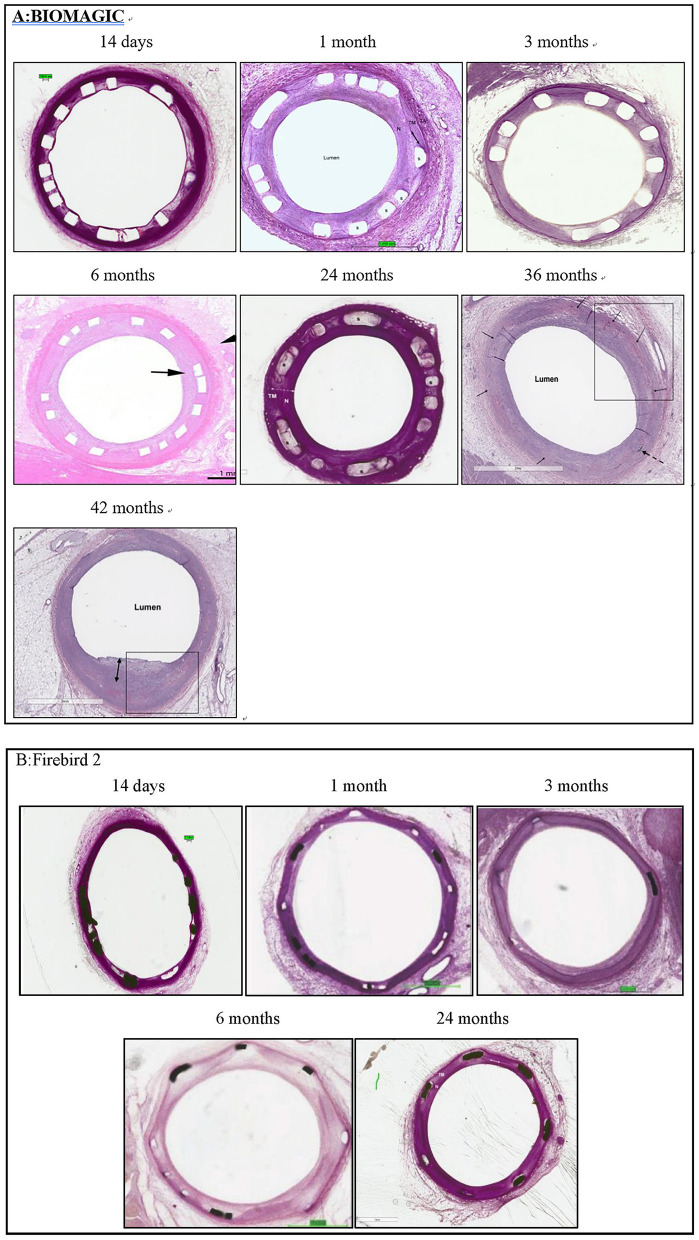
Representative histological images histomorphological analysis was performed by conventional paraffin histological technique and hematoxylin eosin stain. **(A)** Typical histological images of Biomagic BVS in porcine coronary artery from 14 days to 42 months. **(B)** Typical histological images of Firebird2 DES in porcine coronary artery from 14 days to 42 months. Firebird2 was not observed by OCT for 36 and 42 months.

Exemplary histological images of Biomagic BVS and Firebird2 DES were showed in Figure 2. For both BVS and DES, the inflammation score was low (<1) for all the time points observed and endothelialization of implanted BVS the DES was completed within 3 and 1 month, respectively.

**Figure 3:**
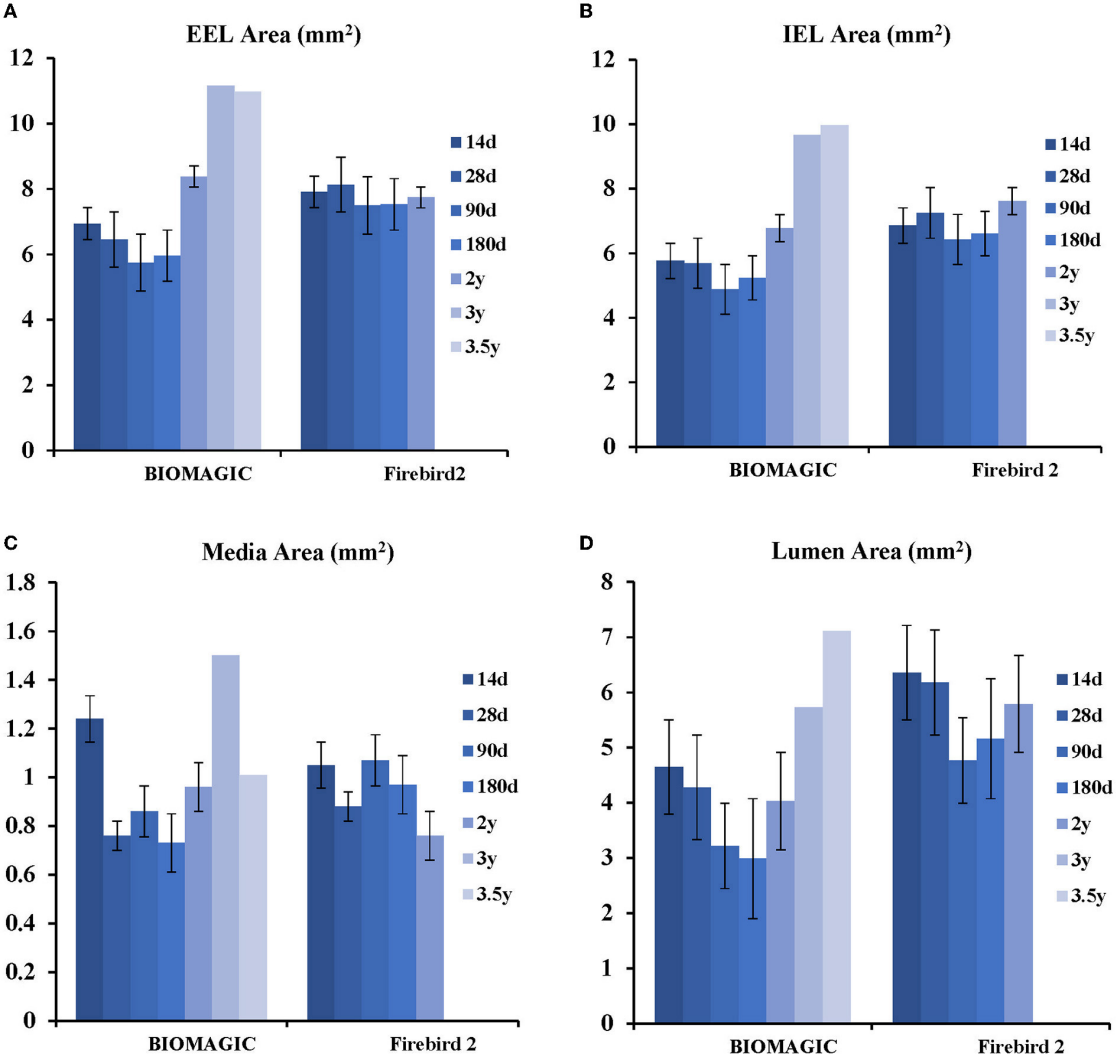
Histological analysis of Biomagic BVS and Firebird2 DES **(A)** comparison curve of EEL. **(B)** Comparison curve of IEL. **(C)** Comparison curve of media area. **(D)** Comparison curve of lumen area.

Morphometric studies (Figure 3, Table 3) showed that post BVS implantation, the EEL area, IEL area, Media area, and lumen area changed with time in a similar fashion: a trend of decreasing started in 1-month, bottomed at 3- to 6-month, and then increased from 24-month to peak at 36- to 48-month. This expansile vascular remodeling in Biomagic BVS was not seen in the Firebird2 DES.

**Table 3:** Histological analysis.

	**14 d**	**1 m**	**3 m**	**6 m**	**24 m**	**36 m**	**42 m**
**EEL, mm^2^**							
Biomagic	6.94 ± 0.78	6.45 ± 0.49	5.75 ± 0.38	5.96 ± 1.23	8.38 ± 0.47	11.16 ± 0.52	10.97 ± 1.07
Firebird2	7.91 ± 0.60	8.13 ± 1.39	7.50 ± 1.06	7.53 ± 1.26	7.74 ± 2.59	NA	NA
*P-value*	0.000	0.000	0.000	0.001	0.751	NA	NA
**IEL, mm^2^**							
Biomagic	5.77 ± 0.57	5.69 ± 0.45	4.88 ± 0.61	5.24 ± 1.14	6.78 ± 2.46	9.66 ± 0.41	9.96 ± 0.93
Firebird2	6.86 ± 0.68	7.25 ± 1.30	6.43 ± 1.08	6.61 ± 1.24	7.62 ± 0.33	NA	NA
*P-value*	0.000	0.000	0.000	0.002	0.659	NA	NA
**Media, mm^2^**							
Biomagic	1.24 ± 0.32	0.76 ± 0.12	0.86 ± 0.39	0.73 ± 0.19	0.96 ± 0.26	1.50 ± 0.15	1.01 ± 0.21
Firebird2	1.05 ± 0.29	0.88 ± 0.24	1.07 ± 0.27	0.97 ± 0.30	0.76 ± 0.14	NA	NA
*P-value*	0.590	0.068	0.004	0.021	0.330	NA	NA
**Lumen, mm^2^**							
Biomagic	4.65 ± 0.55	4.28 ± 0.57	3.22 ± 0.73	2.99 ± 1.06	4.03 ± 2.31	5.73 ± 0.34	7.11 ± 1.64
Firebird2	6.36 ± 0.71	6.18 ± 1.34	4.77 ± 1.14	5.16 ± 1.23	5.79 ± 0.06	NA	NA
*P-value*	0.000	0.000	0.000	0.000	0.334	NA	NA
**% Stenosis**							
Biomagic	19.63 ± 2.27	24.78 ± 8.67	34.85 ± 9.21	42.64 ± 15.92	44.01 ± 11.33	40.66 ± 2.45	29.24 ± 10.27
Firebird2	7.39 ± 3.32	15.11 ± 6.15	26.04 ± 10.54	22.11 ± 9.00	23.98 ± 2.48	NA	NA
*P-value*	0.000	0.001	0.012	0.000	0.044	NA	NA
**Neointimal area, mm^2^**							
Biomagic	1.13 ± 0.11	1.41 ± 0.51	1.67 ± 0.37	2.25 ± 0.81	1.83 ± 0.27	3.93 ± 0.28	2.85 ± 0.74
Firebird2	0.50 ± 0.21	1.07 ± 0.41	1.66 ± 0.76	1.45 ± 0.63	2.75 ± 0.26	NA	NA
*P-value*	0.000	0.037	0.974	0.002	2.306	NA	NA
**Injury score**							
Biomagic	0.11 ± 0.22	0.11 ± 0.32	0.42 ± 0.84	0.50 ± 0.50	0.86 ± 0.71	1.00 ± 0.00	1.00 ± 0.00
Firebird2	0.02 ± 0.07	0.04 ± 0.10	0.36 ± 0.58	0.60 ± 0.78	0.41 ± 0.23	NA	NA
*P-value*	0.103	0.379	0.809	0.664	0.417	NA	NA
**Inflammation**							
Biomagic	0.95 ± 0.27	0.35 ± 0.31	0.46 ± 0.75	0.59 ± 0.56	0.92 ± 0.54	0.47 ± 0.08	0.77 ± 0.20
Firebird2	0.47 ± 0.38	0.16 ± 0.27	0.34 ± 0.67	0.34 ± 0.48	0.06 ± 0.09	NA	NA
*P-value*	0.000	0.055	0.614	0.161	0.064	NA	NA
**Stent struts apposed to media**							
Biomagic	100 ± 0%	89 ± 32%	92 ± 21%	95 ± 11%	100 ± 0%	100 ± 0%	100 ± 0%
Firebird2	100 ± 0%	99 ± 4%	90 ± 22%	80 ± 31%	100 ± 0%	NA	NA
*P-value*	NA	0.121	0.383	0.632	NA	NA	NA
**Stent struts covered by neointima**							
Biomagic	52 ± 39%	83 ± 33%	100 ± 0%	100 ± 0%	100 ± 0%	100 ± 0%	100 ± 0%
Firebird2	85 ± 33%	99 ± 3%	100 ± 0%	100 ± 2%	100 ± 0%	NA	NA
*P-value*	0.391	0.058	NA	0.395	NA	NA	NA
**Stent struts covered by endothelium**							
Biomagic	55 ± 38%	83 ± 33%	100 ± 0%	100 ± 0%	100 ± 0%	100 ± 0%	100 ± 0%
Firebird2	85 ± 33%	100 ± 2%	100 ± 0%	100 ± 0%	100 ± 0%	NA	NA
*P-value*	0.443	0.022	NA	NA	NA	NA	NA

*Biomagic indicates Biomagic rapamycin-eluting bioresorbable coronary scaffold system. P-value for Biomagic vs. Firebird2 at each time point. d, day; m, month*.

### Pharmacokinetics

**Figure 4:**
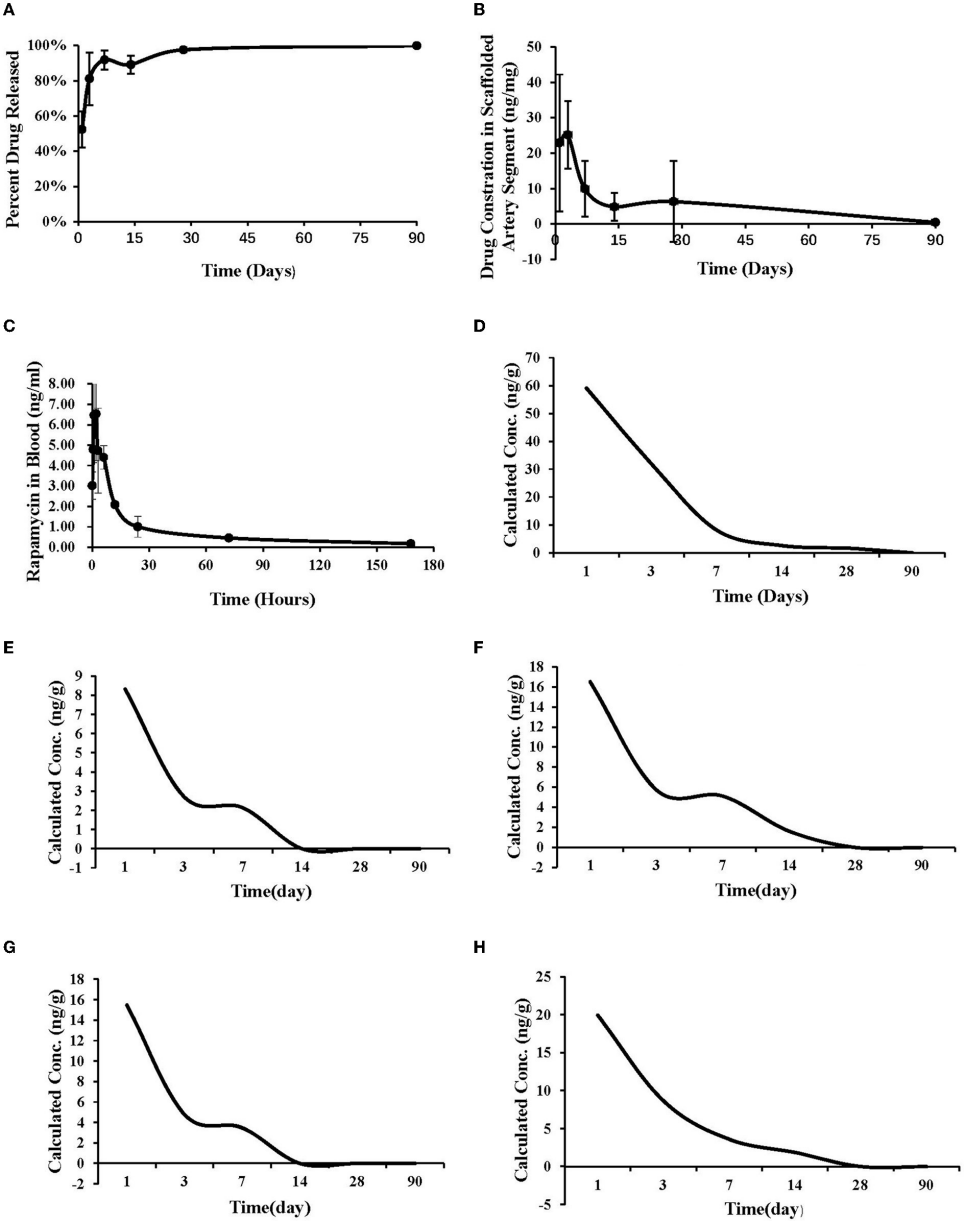
Pharmacokinetic study. **(A)** The drug release curve. **(B)** The drug concentration in the implanted vascular segment. **(C)** The drug concentration in the whole blood after implantation. **(D)** The drug metabolism curve of heart. **(E)** The drug metabolism curve of liver. **(F)** The drug metabolism curve of spleen. **(G)** The drug metabolism curve of lung. **(H)** The drug metabolism curve of kidney.

Summary of studied animals and implanted materials for pharmacokinetics was showed in Table 4. Pharmacokinetic analysis following the BVS implantation was summarized in Figure 4. Sirolimus release from the scaffold was gradual and almost completed in 30 days; the drug concentration in the scaffolded vessel segment was high at the beginning and then gradually reduced to below detectable at 90 days, and at 28 days, the tissue sirolimus level was above the minimal effective therapeutic level (2 ng of sirolimus/mg of tissue). The whole blood sirolimus level was highest at the beginning, then dropped to below 2 ng/ml within 24 h. Sirolimus levels in vital organs (heart, liver, spleen, lung and kidney) were highest immediately after BVS implantation, then decreased quickly to undetectable levels in 14–28 days.

**Table 4:** Summary of studied animals and implanted materials for pharmacokinetics.

	**1 d**	**3 d**	**7 d**	**14 d**	**1 m**	**3 m**	**Total**
Pigs	3	4	3	3	3	4	20
Implants (Biomagic)	6	6	6	6	6	6	36

*Biomagic indicates Biomagic rapamycin-eluting bioresorbable coronary scaffold system. d, day; m, month*.

## Discussion

The principal findings of this current preclinical study are as follows: (1) QCA, OCT, and histomorphology showed that the lumen diameter and lumen area in Biomagic group were smaller than those of Firebird2 group during the early period of the study (<6–12 months), but gradually increased and exceeded the Firebird2 group with longer follow-up. (2) Histomorphology revealed that late vessel enlargement and positive remodeling was observed in Biomagic group, without concurring scaffold malposition. However, this phenomenon was not observed in Firebird2 group. (3) The vascular injury score and inflammation (both <1 point) were observed in Biomagic, which no statistically difference from those of Firebird2. (4) Struts of Biomagic BVS and Firebird2 DES were totally covered by endothelium at 3 months and 1 month respectively. (5) Coronary tissue sirolimus concentrations post Biomagic BVS implantation remained above the 2 ng/mg (minimal therapeutically level) after 28 days.

In the 37°C simulation trial *in vitro*, the radial strength of Biomagic scaffold at 6 months *in vitro* is higher than 140 kPa. During this animal study, OCT showed that mean scaffold diameter of Biomagic maintained a mildly steady growth trend, and no scaffold collapse was observed, indicating that the design and structure of current BVS could provide adequate radial force over time. Furthermore, both intravascular imaging and histopathology demonstrated that lumen diameter in Biomagic was smaller than in Firebird2 during the early period of the study, but gradually increased with time after 12 months. One reason may be because the animal grew larger, so did its heart and coronary artery. Other mechanisms of positive remodeling of vessel may also be contributing to the phenomenon. Previous studies have documented that late luminal enlargement and expansive remodeling following implantation of BVS, but not DES, in both preclinical (13–15) and clinical studies (16). A multivariate analysis of the ABSORB II randomized trial indicated that use of the Absorb BVS, female sex, balloon-artery ratio >1.25, expansion index ≥0.8, previous PCI, and higher level of low-density lipoprotein cholesterol were independent predictors of expansive remodeling (16, 17). Therefore, although the neointimal area in the Biomagic group was somewhat larger as compared with the DES group, the late luminal enlargement and expansive remodeling that the BVS group gained offset the neointimal growth, resulting in its anti-restenosis effectiveness.

Previous studies have confirmed that permanent presence of metallic devices, either bare metal stents (BMS) or DES, continuously exert pressure against the vessel wall, causing vascular injury and inflammatory responses, which are important factors contributing to in-stent restenosis (ISR) (18) and thrombosis (19). Similarly, durable polymer coatings on DES also activate hypersensitive immune and inflammatory responses (20), delaying the process of endothelialization and causing late thrombosis (21). Unlike BMS and DES, BVS only temporarily exists in the vessel and its pressure on vessel wall is reduced drastically after 6 months when it starts to lose its structural integrity. In our studies, Biomagic BVS dropped over 40% and 90% of its original molecular weight at 3 and 6 months, respectively (data not shown) coincide with the initiation of the expansile remodeling in the BVS-implanted vessel. Early removal of outward pressure added upon vessel wall by an implanted device could potentially translate into lower long-term incidence of adverse events (22–25). In the current study, vascular injury and inflammation for Biomagic BVS were mild and not statistically different from those for Firebird2 DES at each time point, which has been reported by other preclinical trials (22). In addition, it has been reported that the rate of polymer degradation affects the degree of inflammatory response (13). Thus, with the degradation and resorption of polymer, inflammation would gradually decrease, leading to lower risk of ISR and thrombosis. In this study, struts of Biomagic was totally covered by endothelium at 3 months, while endothelialization for Firebird2 completed at 1 month. However, no serious adverse events (luminal thrombosis and ISR) occurred in Biomagic BVS group, confirming the safety and efficacy profile of Biomagic BVS suitable for human clinical trials.

## Study limitation

(1) Vascular responses to the devices in healthy swine coronary arteries are probably different from those in diseased patient arteries, which may limit the ability to exert the current findings directly to clinical outcomes in humans. (2) The experimental swine were relatively young at the beginning of the trial. As the swine get older, vascular lumen diameter would increase, which becomes a confounding factor causing the observed late vessel enlargement. (3) This study was not a serial observational trial so that data of vascular changes cannot be recorded continuously in one swine. (4) Inflammatory response in swine coronary arteries after stenting may be different from that in human arteries, because no obvious inflammation was present at the time of stent implantation in swine coronary arteries. (5) The current study used different lengths of stent/scaffold, which may have affected the comparison of the vascular response between them. (6) Angiographic assessment had limited accuracy as compared with histological analysis and intravascular imaging. (7) Due to the difficulties in separating implanted BVS from coronary tissues, the polymer molecular weight changes over time could not be measured with accuracy. (8) No scanning electromicroscopy (SEM) was performed on implanted scaffolds, thus lacking a microscopic assessment of endothelialization. (9) There was no further evaluation of the effect of rapid drug elution in the trial group, such as based on fibrin levels.

## Conclusions

Biomagic demonstrates comparable long-term safety to Firebird2 in porcine coronary arteries with mild to moderate inflammation. Although Biomagic was associated with greater percent stenosis relative to Firebird2 within 36 months, expansile remodeling was observed after 12 months in Biomagic with significantly greater lumen area at ≥36 months. Scaffold resorption is considered complete at 36 months.

## Data availability statement

The original contributions presented in the study are included in the article/supplementary material, further inquiries can be directed to the corresponding author.

## Ethics statement

The animal study was reviewed and approved by Gateway Medical Innovation Center.

## Author contributions

RN-l, YM, JL, and YH contributed to conception and design of the study. FN performed the statistical analysis. YL wrote the first draft of the manuscript. FN, NT, BZhe, and BZha wrote sections of the manuscript. All authors contributed to manuscript revision, read, and approved the submitted version.

## Funding

This work was supported by the developer of Shanghai Biomagic Medical Devices Company Ltd.

## Conflict of interest

Authors RN-l, FN, NT, and YM are employed by Shanghai Biomagic Medical Devices Company Limited. This study received funding from Shanghai Biomagic Medical Devices Company Ltd. The funder had the following involvement with the study: the design and development of the bioabsorbable scaffold. The remaining authors declare that the research was conducted in the absence of any commercial or financial relationships that could be construed as a potential conflict of interest.

## Publisher's note

All claims expressed in this article are solely those of the authors and do not necessarily represent those of their affiliated organizations, or those of the publisher, the editors and the reviewers. Any product that may be evaluated in this article, or claim that may be made by its manufacturer, is not guaranteed or endorsed by the publisher.

## Abbreviations

EEL: external elastic lamina
IEL: internal elastic lamina
PLLA: poly-L-lactide
BVS: bioabsorbable vascular scaffold(s)
DES: drug-eluting stent(s)
BMS: bare-metal stent(s)
DAPT: dual-antiplatelet therapy
MI: myocardial infarction
MACE: major adverse cardiac event(s)
ST: scaffold thrombosis
PSP: pretreatment, sizing, post-dilation
PCI: percutaneous coronary intervention
SES: sirolimus-eluting stent(s)
TLF: target lesion failure, defined as cardiac death
TV-MI: target vessel myocardial infarction
ID-TLR: ischemia-driven target lesion revascularization
TLR: target lesion revascularization, defined as any repeat revascularization procedure (percutaneous or surgical) of the original target lesion site, including the stent and within 5 mm of the proximal and distal stent margins
TVF: target vessel failure
TV-MI: defined as cardiac death
TVR: target vessel revascularization
OCT: optical coherence tomography
QCA: quantitative coronary angiography
MLD: mean lumen diameter.
