# Supplementary material for: Five-year comparative study of thin-strut rapamycin-eluting bioabsorbable scaffold with metallic drug-eluting stent in porcine coronary artery
Source: Front Cardiovasc Med. 2022 Jul 22;9:938519. doi: 10.3389/fcvm.2022.938519 (PMC9355424; doi:10.3389/fcvm.2022.938519)
Supplement: Supplementary file 1 [file Data_Sheet_1.pdf]

10.3389/fcvm.2022.938519

Supplementary Materials

Table 1 | Summary of Studied Animals and Implanted Devices

	14 d	1 m	3 m	6 m	12 m	18 m	24 m	30 m	36 m	42 m	48 m	54 m	60 m
Pigs	10	9	9	9	13	7	13	3	10	2	2	2	1
Implants													
Biomagic	8	9	9	9	11	7	13	4	9	2	2	2	1
Firebird2	9	9	9	9	8	6	5	4	5		1	1	1
QCA													
Biomagic	8	9	9	9	3	7	13	5	9	2	2	2	1
Firebird2	9	9	9	9	2	6	5	4	5		1	1	1
OCT	10	9	9	9	3	7	9	3	8	2	2	2	
Biomagic	8	8	9	9	3	6	10	4	9	2	2	2	
Firebird2	9	9	9	9	2	6	4	3	5		1	1	
Light microscopy													
Biomagic	8	8	9	9			3		1	2			
Firebird2	8	9	9	9			1						

Biomagic indicates Biomagic rapamycin-eluting bioresorbable coronary scaffold system. Firebird2 indicates Firebird2 rapamycin-eluting cobalt-based alloy stent. d- day, m- month.

Table 2 | Models of Implants at each time points

	**14 d**	**1 m**	**3 m**	**6 m**	**12 m**	**18 m**	**24 m**	**30 m**	**36 m**	**48 m**	**54 m**	**60 m**
Biomagic 3.0×08 mm		5	4	2								
Biomagic 3.0×12 mm		3	4	5	2		2					
Biomagic 3.0×18 mm	8				13	7	10	4	9	5	1	1
Firebird 2 3.0×13 mm		10	9	8								
Firebird 2 3.0×18 mm	10				9	6	5	4	5	2		1

Biomagic indicates Biomagic rapamycin-eluting bioresorbable coronary scaffold system. d- day, m- month.
